# Assessing the Impact of Methodological Assumptions on Estimated Age of Disease-Causal Human Papillomavirus Infection in US Females

**DOI:** 10.1093/ofid/ofaf548

**Published:** 2025-09-10

**Authors:** Alhaji Cherif, Xuedan You, John Cook, Miriam Reuschenbach, Ya-Ting Chen, Craig Roberts, Ruifeng Xu

**Affiliations:** Biostatistics and Research Decision Sciences (BARDS), Merck & Co., Inc., Rahway, New Jersey, USA; Value and Implementation (V&I), Merck & Co., Inc., Rahway, New Jersey, USA; Modeling and Adaptation, CHEORS, Chalfont, Pennsylvania, USA; Global Medical & Scientific Affairs, MSD Sharp & Dohme GmbH, Munich, Germany; Value and Implementation (V&I), Merck & Co., Inc., Rahway, New Jersey, USA; Value and Implementation (V&I), Merck & Co., Inc., Rahway, New Jersey, USA; Biostatistics and Research Decision Sciences (BARDS), Merck & Co., Inc., Rahway, New Jersey, USA

**Keywords:** cervical cancer, cervical intraepithelial neoplasia, disease-causal HPV infection, human papillomavirus, vaccination

## Abstract

We adapted a previous discrete event simulation model to assess the impact of varying time distributions from disease-causal human papillomavirus (HPV) infection to CIN2+ onset on the estimated age distribution of disease-casual infection in the United States. Differences in these assumptions had minimal effect on estimates, underscoring the importance of expanded HPV vaccination.

Human papillomavirus (HPV) is a significant cause of various cancers, including cervical cancer and its precursor, high-grade cervical intraepithelial neoplasia (CIN2+). Despite efforts to control these diseases, cervical cancer continues to pose a substantial burden in the United States (US) [1, 2]. In the United States, HPV vaccination is available for girls and boys aged 11–12, with catch-up vaccination available for women and men through age 26 and shared clinical decision-making for adults aged 27–45 [3]. Expanding HPV catch-up vaccinations to women above age 26 presents an opportunity to further reduce the burden of HPV-related cervical diseases. However, understanding the natural history of HPV infection, particularly estimating the age distribution of disease-causal infection, is crucial in understanding the epidemiology of HPV-related diseases and for designing effective public health strategies.

Different studies have reported varied median ages of disease-causal HPV infection in US women, ranging from 19.1 to 34.0 years (or 25.1 to 49.9 years in the presence of imperfect screening compliance) [4, 5]. While the median age of causal cervical HPV infection reported by Prabhu et al [5] (23.9 years) was similar to that obtained by Burger et al [4], the impact of the assumed age distribution and reliance on censored clinical trial data to estimate the time between infection and disease onset, and on the median age estimation has yet to be explored. Clinical trials investigating HPV infection and its associated diseases are subject to limitations that may lead to biased estimates and inferences about age distribution. Here, we account for these factors to investigate their impacts in estimating reliable real-world age of causal HPV infection in US females.

## METHODS

We adapted a previous discrete event simulation model, involving 1000 women, to estimate the age of causal HPV infection and progression to CIN2+ diagnosis [5]. The model tracks women through 3 health stages while undergoing screening—HPV infection, CIN2+ onset, and CIN2+ diagnosis—and simulates age at causal HPV infection, age at CIN2+ onset, and age at CIN2+ diagnosis. The cumulative age distribution curve of real-world CIN2+ diagnosis is shifted backward by an optimized time from HPV infection to CIN2+ diagnosis (offset) to predict the age of causal infection.

Data from vaccine trials, screening registries, and age-specific CIN2+ diagnoses from surveillance registries were used [6–8]. The time from causal infection to CIN2+ onset was estimated using data from the VIVIANE bivalent (2vHPV) and the FUTURE I quadrivalent (4vHPV) HPV vaccine trials [6, 7]. The 2vHPV trial showed that ∼50% of infections progressed to CIN2+ within 1.5 years, while 90% of infections cleared within 4 years. The placebo arm of the 4vHPV trial showed that 70%–100% of infections that progressed to CIN2 or CIN3 did so within 1–2 years. Median time from onset of persistent HPV infection to CIN2+ was estimated to be ≤1.4 years using a gamma distribution with a 6-month offset. The estimated mean (95% CI) and median (95% CI) time from onset of persistent HPV infection to CIN2+ onset were 1.5 (95% CI: 1.4–1.6) years and 1.2 (95% CI: 1.1–1.3) years, respectively.

Screening frequency data from the 2008 New Mexico HPV Pap Registry were used, with exponential distribution utilized to align the age-specific proportion of women screened per year [8]. To obtain the optimal model offset (ie, the duration between causal HPV infection and CIN2+ diagnosis), we calibrated the model by assessing the χ^2^ goodness-of-fit criteria between the predicted and observed real-world CIN2+ diagnosis data. The optimal offset was then used to predict the age distribution of causal HPV infection. We performed 100 different replicates to compute the mean and 95% CI of both predicted age distribution of CIN2+ diagnosis and causal HPV infection, and 10 replicates for the rest of the analyses. We fitted cubic splines to the estimated cumulative distributions using penalized inverse variance–weighted least squares with the smoothing parameter selected by the generalized cross-validation method.

We assessed the impact of 2 novel factors in this analysis: (1) different distributions of time to CIN2+ onset and (2) censoring of source data from the clinical trials. For the first factor, we compared gamma and exponential distributions of time from causal infection to CIN2+ onset in terms of the model-estimated age at CIN2+ diagnosis and age at causal infection. For the second factor, we assessed the impact of censoring in the FUTURE I trial by adjusting the cumulative probability of progression to CIN2+. Because the FUTURE I clinical trials were censored after 3 years [6], we explored the impact of time from HPV infection to CIN2+ onset on the distribution of age of acquisition of causal HPV infection and CIN2+ diagnosis. Since we did not have information on the proportion of patients with persistent HPV infection or CIN1 who would have progressed to CIN2+ beyond 3 years, we made several assumptions about the proportions and recalculated the data accordingly. The proportions were varied from 0% (ie, all remaining patients clearing their infection) to 20% (ie, 20% of these patients progressing to CIN2+, and 80% clearing their infection without progressing to CIN2+). By adjusting the original data based on the different assumptions, we fitted each to a gamma distribution to obtain the distribution for the time from infection to CIN2+ onset.

This decision analytical model study did not require institutional review board review because we used aggregated data without identifiable personal health information and simulation-based research, per Common Rule 45 CFR §102 (e) [9]. This study followed the Strengthening the Reporting of Empirical Simulation Studies reporting guideline [10].

## RESULTS

**Figure 1.**
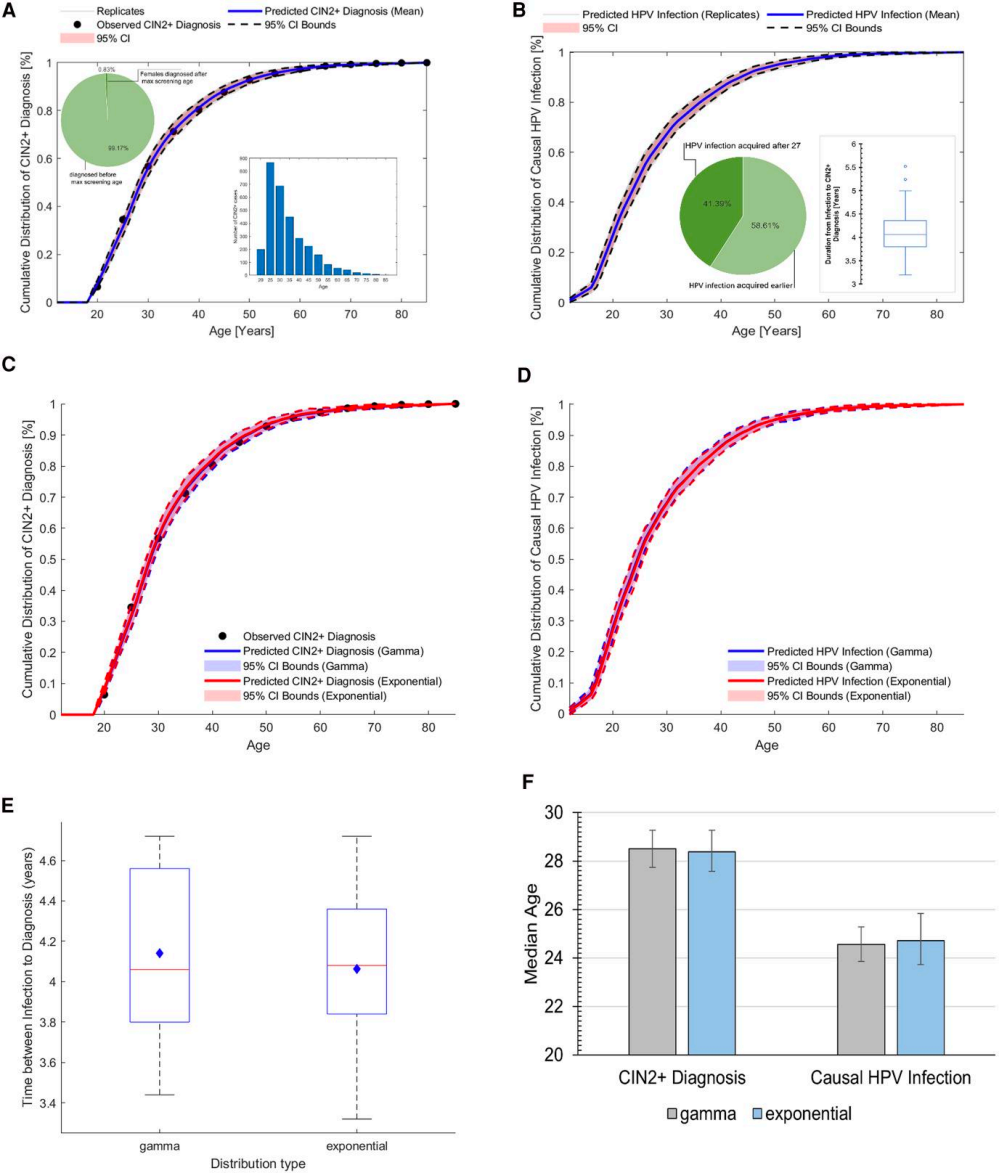
*A*, Age distribution of CIN2+ diagnosis among women attending screening in 2008, with insets showing the proportions diagnosed before and after the maximum screening age, as well as the age distribution of CIN2+ cases. The estimated median age of CIN2+ diagnosis is 28.63 (95% CI: 29.36–27.93), which was within 0.5% of 28.49, the empirical (observed) median age of CIN2+ diagnosis. *B*, Distribution of predicted age of acquisition of causal HPV infection, with a pie inset showing the proportion of HPV infections acquired before and after age 27, and a boxplot inset illustrating the predicted distribution of time between acquiring causal HPV infection and CIN2+ diagnosis. *C* and *D* The impact of assumed distribution (gamma and exponential distributions) of the time from causal HPV infection to CIN2+ onset on (*C*) age at CIN2+ diagnosis and (*D*) age at causal HPV infection. The 2 assumed distributions yielded similar results. *E* and *F*, The impact of assumed distribution for time from causal HPV infection to CIN2+ onset on the time from infection to (*E*) CIN2+ diagnosis and (*F*) the median age at causal infection and CIN2+ diagnosis.

Figure 1 summarizes the results of our model, with Figure 1*A* and 1*B* showing the age distribution of CIN2+ diagnosis and disease-causing HPV infection, respectively. The estimated median age for acquiring causal HPV infection was 24.9 years (95% CI: 23.8–25.9), consistent with previous findings [1, 2] (Figure 1*A* and 1*B*). Approximately 41.4% (95% CI: 37.6–45.2) of causal HPV infections were acquired after the age of 27, aligning with the 42.7% reported in Prabhu et al. The optimal offset was 4.1 years (95% CI: 4.0–4.2) (see Figure 1*B*, inset).

Figure 1 *C* and 1*D* depict the impact of alternative distributions for time from causal infection to CIN2+ onset on the age distribution of causal infection. We observed no statistically significant difference in the median time from causal infection to CIN2+ diagnosis when using gamma versus exponential distribution for time between infection and CIN2+ onset (*P* = .9875, Figure 1*E*), and hence no effect on the median age of disease-causing HPV infection (Figure 1*F*).

**Figure 2.**
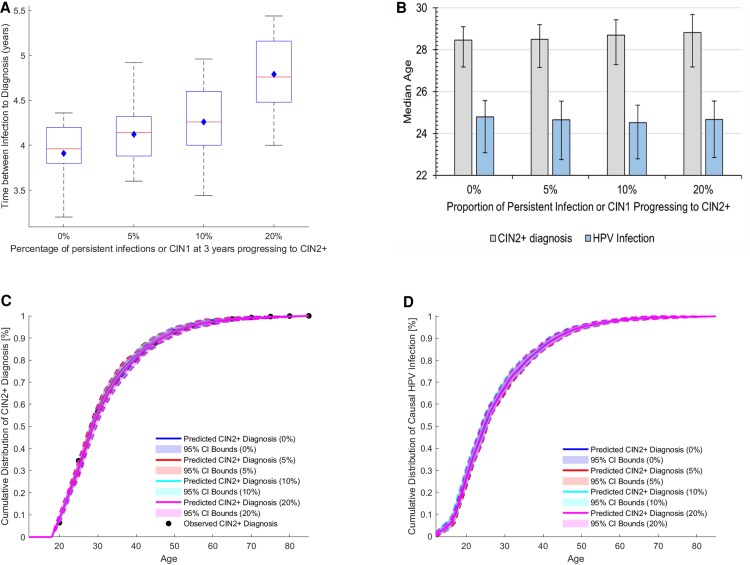
*A* and *B*, The impact of the proportion of subjects with persistent HPV infection or CIN1 progressing to CIN2 (0%, 5%, 10%, or 20%) after the 3-year censored FUTURE I clinical trial date on (*A*) the estimated time from causal HPV infection to CIN2+ diagnosis, and (*B*) the corresponding median age of CIN2+ diagnosis and causal HPV infection. In the box plots, the line inside the box represents the median, and the diamond denotes the mean. In both (*A* and *B*), the error bars show the 95% CI. *C* and *D*, Impact of the proportion of subjects with persistent HPV infection or CIN1 progressing to CIN2 (0%, 5%, 10%, or 20%) after the 3-year censored FUTURE I clinical trial date on (*C*) the fitting of CIN2+ diagnosis and (*D*) estimated causal HPV infection, respectively. The shaded area of the distribution curves corresponds to the 95% CI, with the dashed line representing the 95% CI upper and lower bounds across the 100 best-fitted simulations. The solid dot denotes the observed data. The solid line corresponds to the average of the best-fitted simulation replications.

Figure 2 *A* illustrates the distribution of time from HPV infection to CIN2+ diagnosis, as a function of the unknown proportion of patients with persistent infection or CIN1 at the end of the trial who subsequently progress to CIN2+ onset after the trial. Although both the mean and median time between infection and diagnosis were found to significantly increase with the proportion of persistent disease (*P* < .0001), the impact on the estimated median age at disease diagnosis and causal infection was marginal (see Figure 2*B*), and the differences were not statistically significant (*P* = .2032). Figure 2*C* and 2*D* show the corresponding age distributions of CIN2+ diagnosis and of causal HPV infection.

## DISCUSSION

Our study contributes valuable insights to the methodology of estimating age distribution for causal HPV infection. The median age for acquiring causal HPV infection was 25 years, consistent with findings from Burger et al [4] and Prabhu et al [5]. The estimated time between causal HPV infection and observed CIN2+ diagnosis was 4 years, suggesting HPV-related diseases begin to manifest shortly after infection occurs during early adulthood. Approximately 41% of causal HPV infections were acquired after the age of 27, in agreement with the reported 42.7% of Prabhu et al [5], highlighting the importance of targeting individuals beyond the typical age range for preventive interventions. Mid-adult women remain at risk, as natural immunity does not provide complete protection. New infections may be acquired from the same sexual partner, new sexual partners, or autoinoculation from other infected anatomical sites.

We found no significant difference between using gamma or exponential distribution for estimating the time from infection to CIN2+ onset. The impact of censoring on the age distribution of disease-causing infection was minimal in the context of the United States. However, these findings may be influenced by the limitations posed by variations in the sensitivity of CIN2+ diagnosis and the frequency of screening at various ages in different places.

The modified model retains the strengths of the original Prabhu et al model [5] while allowing for exploration of different assumptions regarding the natural history of the disease, for example, different times from infection to disease onset and different proportions of persistent infection. Although the model does not differentiate between types of CIN2+ and HPV genotypes, future studies should explore genotype-specific trajectories given their distinct oncogenic potentials and progression timelines. Stratifying the age distribution of CIN2+ diagnosis and time from infection to CIN2+ onset by genotypes and lesion types could potentially overcome this limitation in future research.

## References

[1] Senkomago V, Henley SJ, Thomas CC, Mix JM, Markowitz LE, Saraiya M. Human papillomavirus-attributable cancers—United States, 2012–2016. MMWR Morb Mortal Wkly Rep 2019; 68:724–8. 31437140 10.15585/mmwr.mm6833a3 PMC6705893

[2] Van Dyne EA, Henley SJ, Saraiya M, Thomas CC, Markowitz LE, Benard VB. Trends in human papillomavirus-associated cancers—United States, 1999–2015. MMWR Morb Mortal Wkly Rep 2018; 67:918–24. 30138307 10.15585/mmwr.mm6733a2 PMC6107321

[3] Meites E, Szilagyi PG, Chesson HW, Unger ER, Romero JR, Markowitz LE. Human papillomavirus vaccination for adults: updated recommendations of the advisory committee on immunization practices. MMWR Morb Mortal Wkly Rep 2019; 68:698–702. 31415491 10.15585/mmwr.mm6832a3 PMC6818701

[4] Burger EA, de Kok I, Groene E, et al Estimating the natural history of cervical carcinogenesis using simulation models: a CISNET comparative analysis. J Natl Cancer Inst 2020; 112:955–63. 31821501 10.1093/jnci/djz227 PMC7492768

[5] Prabhu VS, Roberts CS, Kothari S, Niccolai L. Median age at HPV infection among women in the United States: a model-based analysis informed by real-world data. Open Forum Infect Dis 2021; 8:ofab111. 10.1093/ofid/ofab111 PMC8653628 34888404

[6] Insinga RP, Perez G, Wheeler CM, et al Incident cervical HPV infections in young women: transition probabilities for CIN and infection clearance. Cancer Epidemiol Biomarkers Prev 2011; 20:287–96. 21300618 10.1158/1055-9965.EPI-10-0791

[7] Skinner SR, Wheeler CM, Romanowski B, et al Progression of HPV infection to detectable cervical lesions or clearance in adult women: analysis of the control arm of the VIVIANE study. Int J Cancer 2016; 138:2428–38. 26685704 10.1002/ijc.29971 PMC4787275

[8] Cuzick J, Myers O, Hunt WC, et al A population-based evaluation of cervical screening in the United States: 2008–2011. Cancer Epidemiol Biomarkers Prev 2014; 23:765–73. 24302677 10.1158/1055-9965.EPI-13-0973 PMC4011954

[9] US Department of Health and Human Services . 45 CFR 46. November 13, 2024; Available at: https://www.hhs.gov/ohrp/regulations-and-policy/regulations/45-cfr-46/index.html. Accessed 28 January 2025.

[10] Monks T, Currie CSM, Onggo BS, Robinson S, Kunc M, Taylor SJE. Strengthening the reporting of empirical simulation studies: introducing the STRESS guidelines. J Simul 2019; 13:55–67.

